# Guanosine Dianions Hydrated by One to Four Water Molecules

**DOI:** 10.1021/acs.jpclett.2c00512

**Published:** 2022-04-05

**Authors:** Samanta Makurat, Qinqin Yuan, Jacek Czub, Lidia Chomicz-Mańka, Wenjin Cao, Xue-Bin Wang, Janusz Rak

**Affiliations:** † Faculty of Chemistry, University of Gdańsk, Wita Stwosza 63, Gdańsk 80-308, Poland; ‡ Physical Sciences Division, Pacific Northwest National Laboratory, Richland, Washington 99352, United States; § Department of Physical Chemistry, Gdańsk University of Technology, Narutowicza 11/12, Gdańsk 80-233, Poland; ∥ BioTechMed Center, Gdańsk University of Technology, Narutowicza 11/12, Gdańsk 80-233, Poland; ⊥ Department of Chemistry, Anhui University, Hefei, Anhui 230601, China

## Abstract

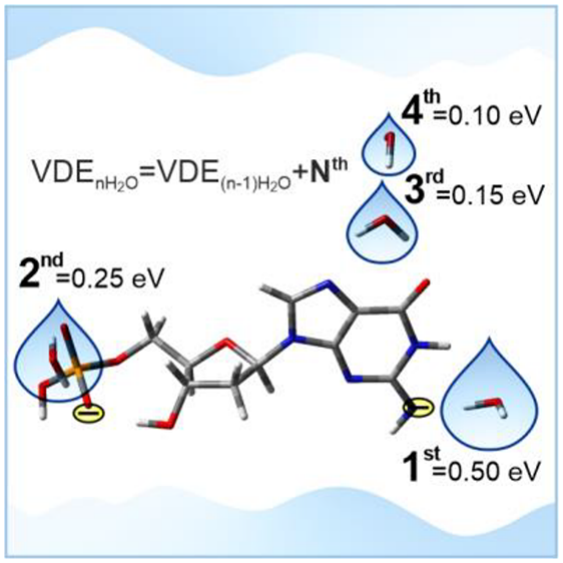

Intermolecular interactions such as those present in molecule···water complexes may profoundly influence the physicochemical properties of molecules. Here, we carried out an experimental–computational study on doubly deprotonated guanosine monophosphate···water clusters, [dGMP – 2H]^2–^·*n*H_2_O (*n* = 1–4), using a combination of negative anion photoelectron spectroscopy (NIPES) with molecular dynamics (MD) and quantum chemical (QM) calculations. Successive addition of water molecules to [dGMP – 2H]^2–^ increases the experimental adiabatic detachment (ADE) and vertical detachment energy (VDE) by 0.5–0.1 eV, depending on the cluster size. In order to choose the representative conformations, we combined MD simulations with a clustering procedure to identify low energy geometries for which ADEs and VDEs were computed at the CAM-B3LYP/6-31++G(d,p) level. Our results demonstrate that the assumed approach leads to sound geometries and energetics of the studied microsolvates since the calculated ADEs and VDEs are in pretty good agreement with the experimental characteristics. The evolution of hydrogen bonding with cluster size indicates the possibility of the occurrence of proton transfer for clusters comprising a larger number of water molecules.

Radiation-induced DNA damage, such as strand breaks, mutations, photolesions, and transcription errors, leading to cancer, has been known for a long time.^1−5^ To clarify the mechanisms of oxidative damage in these processes, numerous spectroscopic and theoretical investigations have been performed to explore the intrinsic geometric and electronic properties for isolated nucleotides, the basic building blocks of DNA molecules.^6−14^ As such, a broad range of knowledge on isolated nucleotides in various charge states, that is, neutral, [dNMP], deprotonated or radical anions, [dNMP – H]^−^/[dNMP]^•–^, and double-deprotonated dianions [dNMP – 2H]^2–^ (N = A, G, C, or T), including their geometric and electronic structures, as well as the mechanisms of indirect and direct electron detachments, has been established.^6,7,14^

The information on isolated nucleotides is crucial to the understanding of the properties of DNA molecules. Nevertheless, their interactions with solvent water molecules remains poorly studied, despite the fact that most ionizing radiation-induced DNA damage processes take place in solution or at interfaces. Such information is especially desired since discrepancies in the properties of DNA or nucleotides in the gas phase and solutions have often been observed. However, experimental characterization of solvated biological molecules is challenging and far from settled. The challenges are related to two issues: (1) what kinds of isomers are generated and (2) how can they be spectroscopically probed; this becomes especially troublesome due to the complexity of hydration and flexibility of biological molecules. As discussed in a very recent article by Garand and co-workers,^15^ the exact ensemble of isomers remains debatable and depends on how they are generated.

Infrared (IR) spectroscopy is a powerful experimental method to probe gas-phase ion molecular structure and has been widely applied. Limitations in this methodology arise, however, with increasing molecular complexity as the number of possible structures increases and the vibrational spectra become more complicated.^15^ Photoelectron spectroscopy (PES) provides another spectroscopic tool to investigate gas-phase ion molecular structures and can directly probe electronic stabilization upon solvation. The obtained vertical and adiabatic detachment energies (VDE, ADE) are sensitive to the local solvation environment. Additionally PES often exhibits salient new spectral features when a charged chromophore changes upon solvation (for example, upon proton transfer, see ref (35)).

It is worth emphasizing that strand breaks of DNA have been proven to occur more often in hydrated DNA than in dry DNA.^16^ Moreover, the studies on nucleotide electron attachment in aqueous solutions have demonstrated different mechanisms from that in the gas phase.^17−22^ The influence of proton transfer involving water molecules seems to be especially interesting. That process is impossible for the isolated nucleotides but may occur in water clusters or in bulk water. As indicated by the ab initio molecular dynamics (AIMD) simulations of Kohanoff et al.,^21^ the protonation of anionic nucleotides in water hinders the O–P bond cleavage (a model of single strand break in DNA), which suggests that native DNA is not sensitive to hydrated electrons. The electron-attached nucleotides are well-known to be exotic,^23,24^ while neutral nucleotides are generally considered protonated and deprotonated in an aqueous solution.

An effective approach to study the underlying mechanism of solvation is to investigate microsolvated clusters so that the effects from interactions between the nucleotides and each of the water molecules can be studied stepwise. For nucleotides, with a nucleobase and a phosphate group, both of which could interact with the H_2_O molecule, the question of which site will preferentially bind to water molecules could be naturally raised. To date, the microsolvated clusters of nucleotides have only been studied via mass spectrometry based kinetic methods and theoretical calculations for singly protonated and deprotonated nucleotides,^25^ while no spectroscopic measurements have been carried out for gaseous microsolvated nucleotide clusters to confirm conformer structures yet. Moreover, doubly deprotonated 2′-deoxynucleoside 5′-monophosphate dianions, which possesses two charged sites to bind to the H_2_O molecule, were recently found to be stable in the gas phase and bring further challenges toward the analysis of binding with H_2_O molecules.^14^

Figure 1 exhibits the 20 K negative ion photoelectron (NIPE) spectra of [dGMP – 2H]^2–^·*n*H_2_O (*n* = 1–4) (red) recorded at 157 nm (7.866 eV) and the comparison to previously recorded [dGMP – 2H]^2–^ (blue^14^) spectrum (for experimental details see section 1.1 of the Supporting Information). The spectra of microsolvated clusters possess similar patterns to that of the [dGMP – 2H]^2–^ anion,^14^ which are composed of a distinct low-intensity band followed by a series of more intense highly congested bands. These bands represent the transitions arising from the ground electronic state of a dianion at a specific low-lying isomer to the ground and multiple electronic excited states of the corresponding singly charged anion, while the experimental ADE and VDE values are determined from the threshold and maximum of the lowest EBE (electron binding energy) band, respectively. Due to the similarities in spectral profile, we mainly focus on this band for each of the spectra of solvated clusters that reflects the ADE and VDE shift. The hydrated clusters exhibit a stepwise blue shift in EBE with increasing number of water molecules due to solvent stabilizations. The incremental ADE or VDE shifts, that is, ADE(*n*)/VDE(*n*) – ADE(*n* – 1)/VDE(*n* – 1), were measured to be 0.50, 0.25, 0.15, and 0.10 eV for *n* = 1, 2, 3, and 4, respectively. The first water molecule results in the strongest stabilization of 0.50 eV, 2 times bigger than that from the second water and 5 times greater compared to the effect of the fourth water molecule.

**Figure 1:**
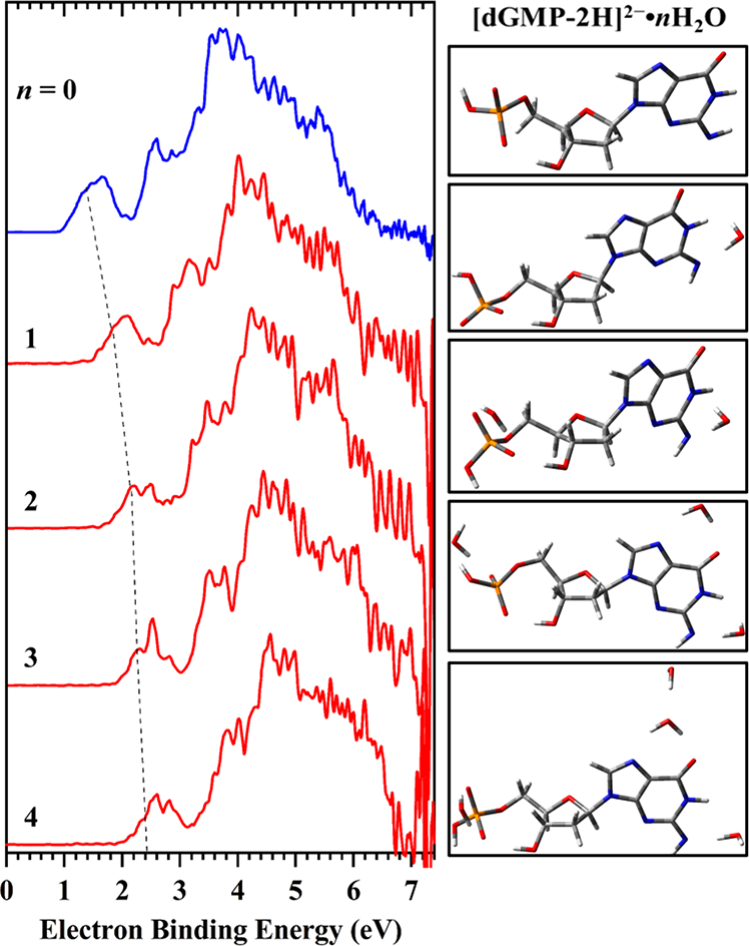
The 20 K NIPE spectra of [dGMP – 2H]^2–^·*n*H_2_O (*n* = 1–4) (red) and [dGMP – 2H]^2–^ (blue, ref (14)) at 157 nm (7.866 eV) (left panel) and the most stable conformations of particular clusters having VDE and ADE characteristics that reproduce the measured values (right panel). The dashed line serves as an eye-guide for the increasing trend of the VDEs as the number of water molecules increases from 0 to 4.

In order to explain the above-mentioned effects at the molecular level, we conducted conformational analysis of the microsolvated [dGMP – 2H]^2–^ anion followed by VDE/ADE calculations for each identified cluster.

Various approaches were used to computationally describe microsolvation effects as this is not a trivial task. Indeed, the search for low energy conformers is an NP-hard (nondeterministic polynomial time hard) problem,^26^ which generally cannot be solved by a brute-force method. An exemplary illustration of this difficulty is a recent work from Boldyrev’s group,^27^ who showed that for a modest discretization of spatial and angular conformational degrees of freedom, the number of initial geometries for a relatively small NO_3_^–^(H_2_O)_12_ hydrate amounts to 10^42^ initial configurations. Thus, chemical intuition,^28^ water density distribution around large fragments of biological importance (e.g., around DNA),^29^ molecular dynamics,^30^ hybrid QM/MM approach,^31^ etc., have been used in the past to solve the conformational problem in a finite time. Here, we propose another general-purpose computational approach, which is a combination of molecular dynamics simulations followed by clustering of similar configurations and their quantum chemical refinement (for computational details, see sections 1.2.1 and 1.2.2 of the Supporting Information). Our novel approach, employing clustering of similar configurations in an ensemble generated by MD simulations, seems to be relatively simple and efficient. Moreover, it results in averaged VDEs and ADEs calculated for individual low energy geometries remaining in good agreement with the experimental values as is demonstrated below.

According to our previous studies, guanosine phosphate deprotonates at the guanine amine and phosphate group, producing dianionic [dGMP – 2H]^2–^ system, where negative charges are maximally separated, that is, they are localized on the phosphate group and on the purine moiety (see Scheme 1).^14^ Therefore, it could be expected that polar water molecules attach to [dGMP – 2H]^2–^, either close to the deprotonated phosphate group or close to the purine ring, interacting with the deprotonated N10 nitrogen. Indeed, we can observe such localization of water molecules in all the optimized [dGMP – 2H]^2–^·H_2_O geometries (see Figure 2). In the dominant conformation, structure one-m23 (with the highest population of 0.71; for details of how the contribution of particular geometries to the equilibrated mixture of conformers was calculated see ref (32) or section 1.2.3 of the Supporting Information) in the ensemble of conformers equilibrated at 298 K (in the following we will denote the population in the ensemble as an equilibrium fraction, *x*_M_), see Table 1, the water molecule is bound via a hydrogen bond to the anionic deprotonated purine amine group (see Figure 2, the framed geometry). The remaining significant structures (*x*_M_ ≥ 0.01) differ either by water localization or by phosphate group and sugar ring conformation. VDE calculated for the dominant structure amounts to 1.70 eV (for VDE and ADE definitions, see section 1.2.4 of the Supporting Information), which is in good agreement with the experimental VDE of 1.80 eV (see Table 1). The weighted average VDE_avg_, calculated for the complete set of optimized structures, is slightly lower than VDE for the dominant structure and amounts to 1.66 eV, which additionally highlights the role of the one-m23 type of geometry in the formation of the experimental PES spectrum.

**Scheme 1:**
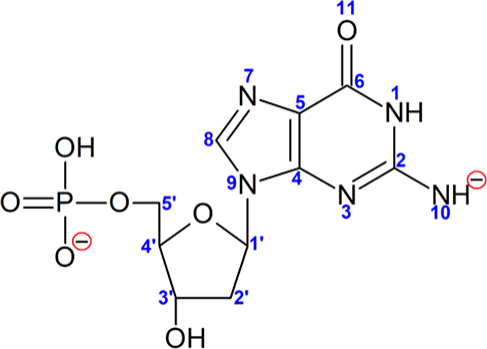
Chemical Formula of [dGMP – 2H]^2–^ along with Atom Numbering

**Table 1:** Relative Gibbs Free Energy Values (Δ*G*, in kcal/mol) and Vertical and Adiabatic Detachment Energy (VDE and ADE, in eV), Calculated for the [dGMP – 2H]^2–^·H_2_O Systems at the CAM-B3LYP/6-31++G(d,p) Level, Characterized by Equilibrium Fraction *x*_M_ ≥ 0.01^a^

name	Δ*G*	VDE	ADE	*x*_M_
manual-9	2.5	1.63	1.20	0.01
one-m1	2.2	1.59	1.31	0.02
one-m2	1.6	1.64	1.33	0.05
one-m5	2.6	1.65	1.36	0.01
one-m7	2.0	1.65	1.33	0.02
one-m9	2.0	1.36	1.18	0.03
one-m12	2.6	1.59	1.31	0.01
one-m14	1.9	1.36	0.97	0.03
one-m17	1.6	1.67	1.35	0.05
one-m18	2.6	1.65	1.36	0.01
one-m20	2.0	1.70	1.38	0.03
**one-m23**	**0.0**	**1.70**	**1.37**	**0.71**
one-m29	2.3	1.33	0.97	0.02
**weighted average**		**1.66**	**1.34**	
experimental values		1.80	1.55	

a Data for the dominant one-m23 geometry, as well as weighted average VDE and ADE, are bolded. Characteristics for all the obtained geometries for singly hydrated [dGMP – 2H]^2–^ are gathered in Table S1 in the Supplementary Information.

**Figure 2:**
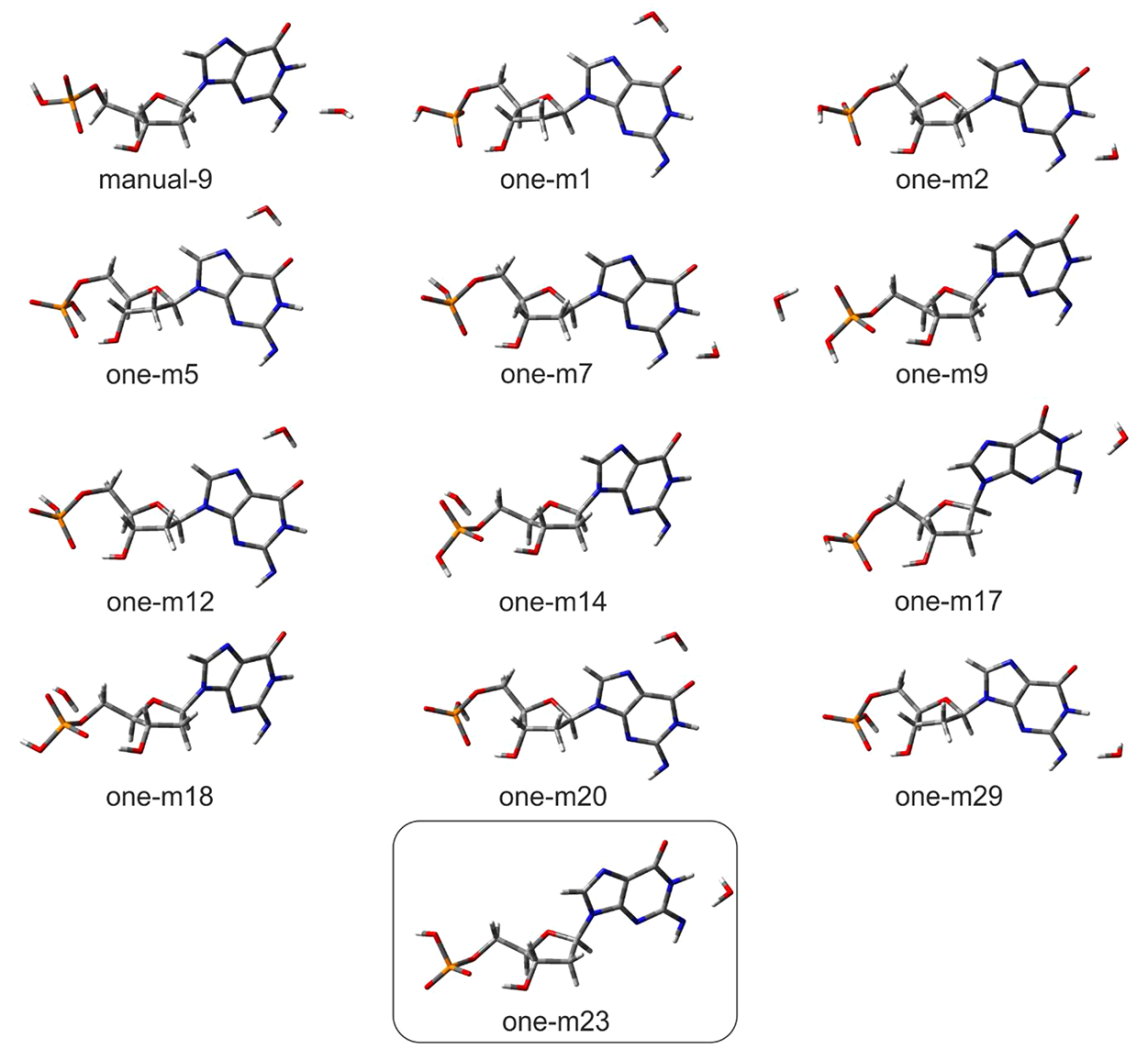
Visualization of dianionic singly hydrated [dGMP – 2H]^2–^·H_2_O structures for which equilibrium fraction amounts to *x*_M_ ≥ 0.01, optimized at CAM-B3LYP/6-31++G(d,p) level,^33,34^ along with their labels. The most stable geometry is shown in the box.

Comparing the results obtained for singly and doubly hydrated [dGMP – 2H]^2–^ systems, one can state that the first water molecule localizes the most willingly near the deprotonated amine group while the second one localizes near the deprotonated phosphate group (cf. the dominant two-m19 and two-m23 structure, see Figure 3). Much less populated are geometries where both waters localize near the purine nucleobase (as in case of two-m3 or two-m24 structures, see Figure 3 and Table 2). The two almost identically stable geometries: two-m19 and two-m23, differing only slightly in the near-phosphate second water location possess the same VDE values (1.81 eV, comparing to 2.05 eV experimental value). As those structures dominate the doubly hydrated dianion ensemble, with the cumulative contribution of 85% (see Table 2), VDE_avg_ estimated for the doubly hydrated system is almost the same as VDE calculated for two-m19 and two-m23, hence underestimated in comparison to the experimental 2.05 eV value. Analyzing the VDEs of the conformers with *x*_M_ ≥ 0.01, one can see that the VDE of two-m24 is similar to the experimental one (Table 2). This two-m24 structure has a different solvation pattern, as the second water molecule localizes near the purine instead of the phosphate group (see Figure 3), which may suggest that in the PES experiment also structures similar to two-m24 are populated.

**Table 2:** Relative Gibbs Free Energy Values (Δ*G*, in kcal/mol) and Vertical and Adiabatic Detachment Energy (VDE and ADE, in eV), Calculated for the [dGMP – 2H]^2–^·2H_2_O Systems at the CAM-B3LYP/6-31++G(d,p) Level, Characterized by Equilibrium Fraction *x*_M_ ≥ 0.01^a^

name	Δ*G*	VDE	ADE	*x*_M_
two-m3	1.4	2.00	1.62	0.04
two-m5	1.9	1.81	1.46	0.02
two-m6	2.0	1.74	1.42	0.01
**two-m19**	**0.0**	**1.81**	**1.32**	**0.46**
two-m22	2.1	1.75	1.42	0.01
**two-m23**	**0.1**	**1.81**	**1.53**	**0.39**
two-m24	1.7	2.07	1.68	0.03
**weighted average**		**1.82**	**1.43**	
experimental values		2.05	1.80	

a Data for the dominant two-19 and two-23 geometries, as well as weighted average VDE and ADE, are bolded. Characteristics for all the obtained geometries for doubly hydrated [dGMP – 2H]^2–^ are gathered in Table S2 in the Supplementary Information.

**Figure 3:**
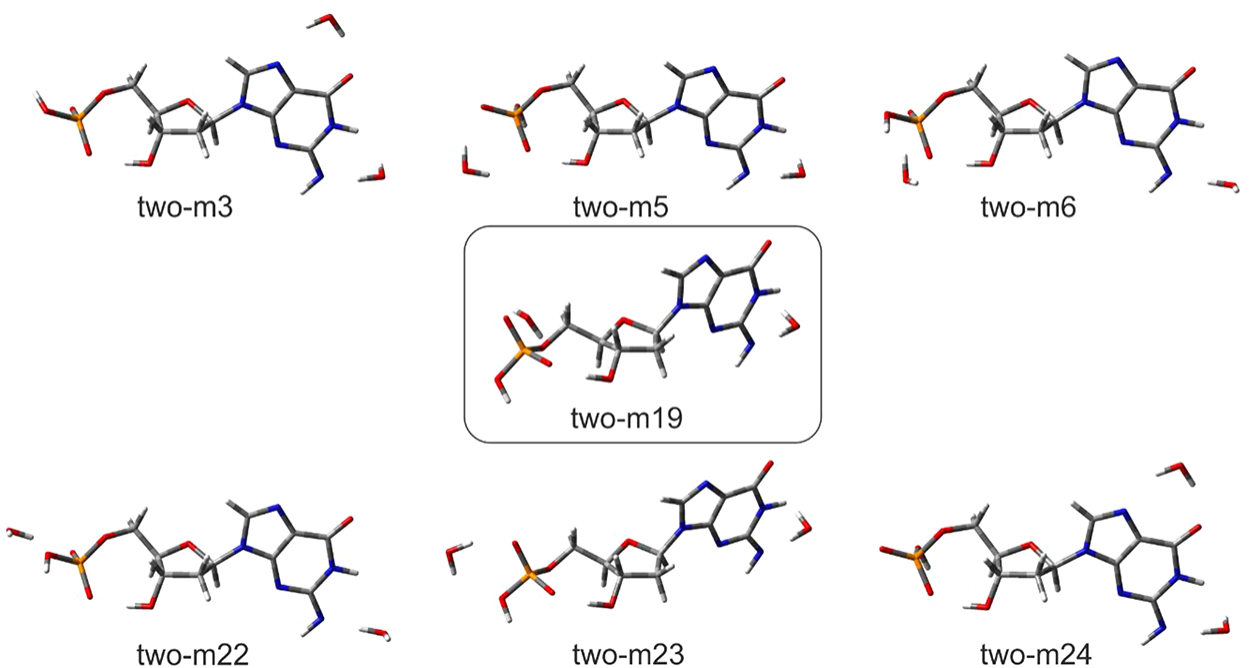
Visualization of dianionic doubly hydrated [dGMP – 2H]^2–^·2H_2_O structures for which equilibrium fraction amounts to *x*_M_ ≥ 0.01, optimized at CAM-B3LYP/6-31++G(d,p) level,^33,34^ along with their names. The most stable geometry is shown in the box.

When the third water molecule is taken into account, we can clearly state that the three most favorable locations for water molecules are as follows: near the amine deprotonated group, near the purine ring, and near the phosphate group (see Figure 4, for example, dominant three-m15 structure). The geometries of all the important structures differ only slightly, mostly in the phosphate group moiety and in the orientation of the near-amine-group water molecule. The only structure that is clearly different from the remaining conformations is three-m21 for which the third water forms a hydrogen bond with the near-amine-group water rather than with the guanosine anion (see Figure 4). However, this structure is rather poorly populated, with *x*_M_ = 0.02 (see Table 3). The calculated VDEs are in very good agreement with the experimental data. Indeed, VDE calculated for the dominant three-m15 structure amounts to 2.07 eV, weighted average VDE_avg_ is equal to 2.11 eV, while the experimental value was measured at 2.20 eV.

**Table 3:** Relative Gibbs Free Energy Values (Δ*G*, in kcal/mol) and Vertical and Adiabatic Detachment Energy (VDE and ADE, in eV), Calculated for the [dGMP – 2H]^2–^·3H_2_O Systems at the CAM-B3LYP/6-31++G(d,p) Level, Characterized by Equilibrium Fraction *x*_M_ ≥ 0.01^a^

name	Δ*G*	VDE	ADE	*x*_M_
three-m1	0.2	2.17	1.76	0.13
three-m2	0.2	2.17	1.76	0.13
three-m3	0.5	2.17	1.77	0.08
three-m4	0.2	2.09	1.72	0.12
three-m5	0.2	2.11	1.72	0.12
three-m6	0.4	2.08	1.71	0.09
three-m13	0.3	2.08	1.71	0.11
**three-m15**	**0.0**	**2.07**	**1.69**	**0.18**
three-m21	1.4	2.37	1.93	0.02
**weighted average**		**2.11**	**1.73**	
experimental values		2.20	1.95	

a Data for the dominant three-m15 geometry, as well as weighted average VDE and ADE, are bolded. Characteristics for all the obtained geometries for triply hydrated [dGMP – 2H]^2–^ are gathered in Table S3 in the Supplementary Information.

**Figure 4:**
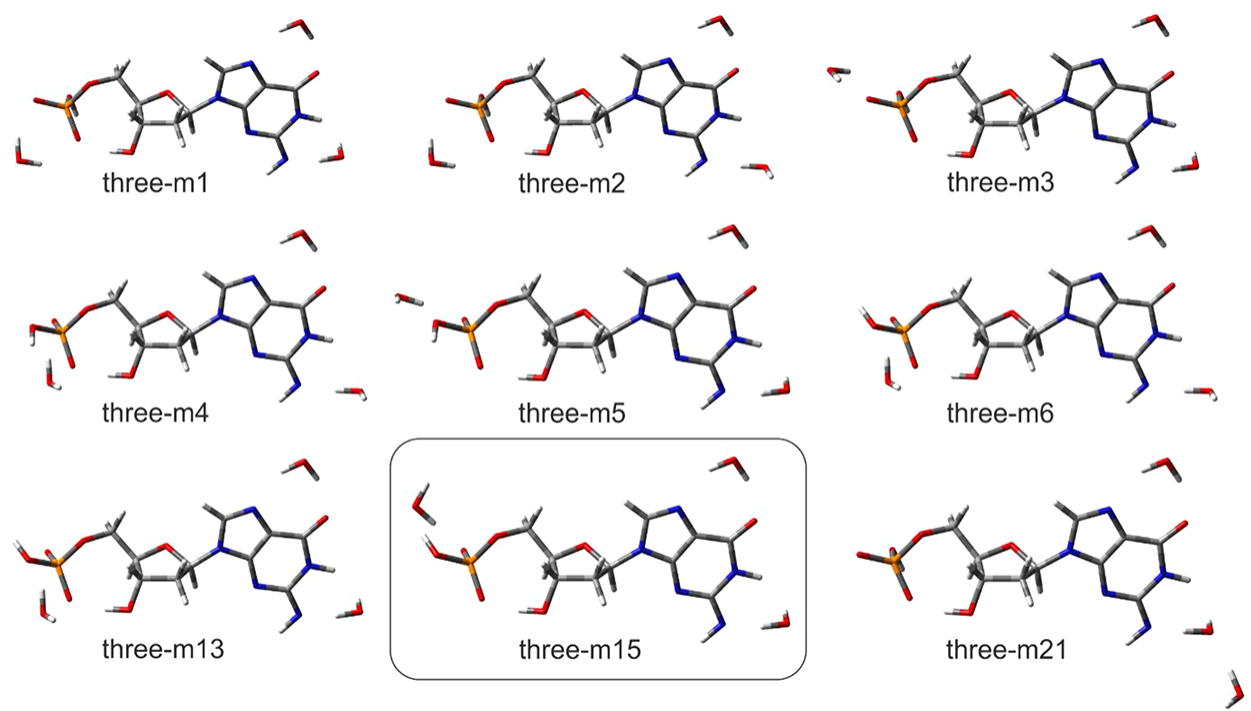
Visualization of dianionic triply hydrated [dGMP – 2H]^2–^·3H_2_O structures, for which equilibrium fraction amounts to *x*_M_ ≥ 0.01, optimized at CAM-B3LYP/6-31++G(d,p) level,^33,34^ along with their names. The most stable geometry is shown in the box.

The analysis of geometries of hydrated guanosine phosphate dianion solvated with four water molecules demonstrates that the three most favorable water locations are the same as those for the triply hydrated complex. These are near the amine group location, near the purine ring location, and near the phosphate group location. The location of the fourth water molecule is not obvious. In fact, it can localize near the phosphate group (see Figure 5, structures four-m1, -m3, -m4, -m5, -m8, -m14, -m15, and -m16) or form hydrogen bonds involving the phosphate group and ribose ring via its C3′-hydroxyl group (see four-m27 structure, Figure 5). The fourth water molecule also makes hydrogen bonds with the other water molecules, not with the guanosine phosphate dianion itself. Such a situation can be observed for the dominant four-m23 structure, where the fourth water binds to the water interacting with the purine ring (see Figure 5). One can also note that solvation by three or more water molecules leads to the stabilization of the 2′-deoxyribose conformation. Indeed, we did not observe any sugar ring conformational changes, as was noted in singly and doubly hydrated systems.

**Figure 5:**
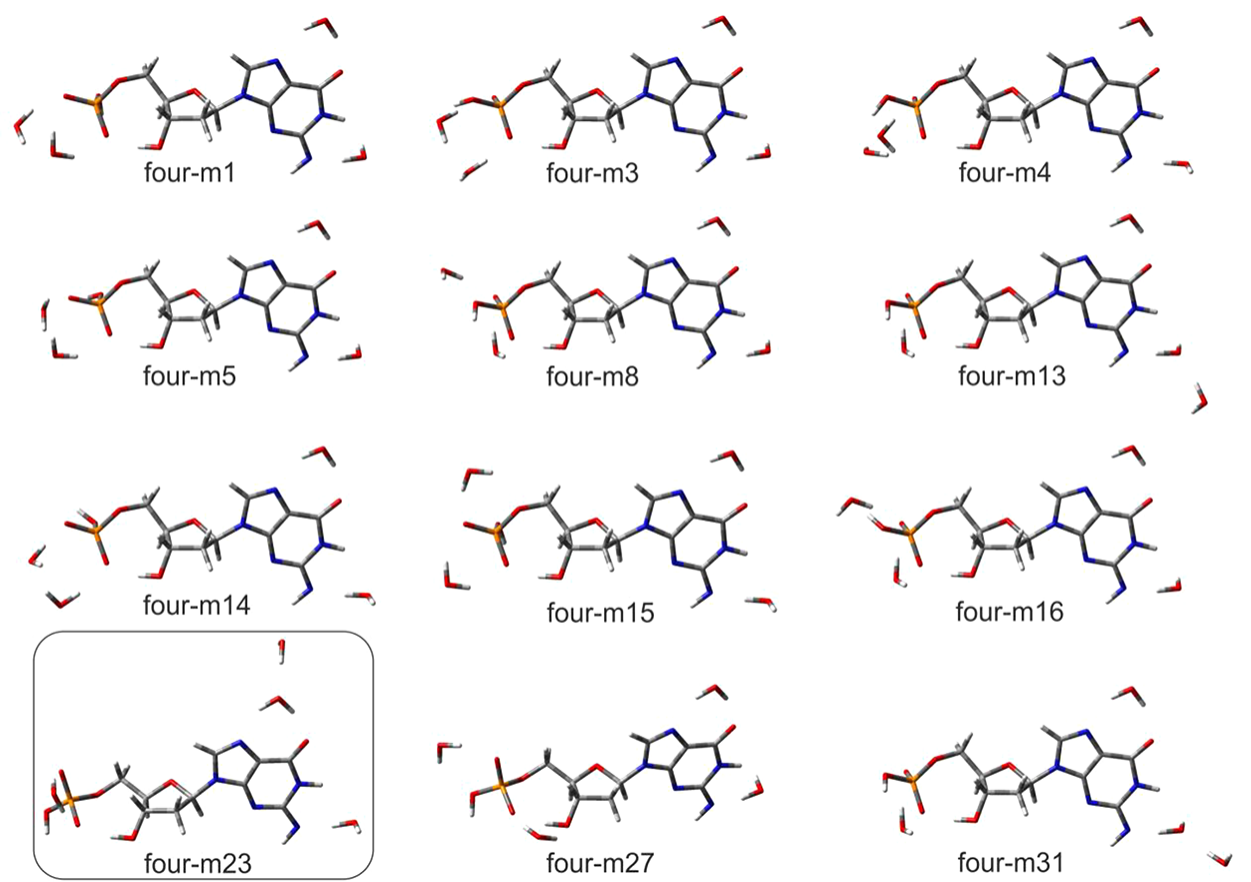
Visualization of dianionic [dGMP – 2H]^2–^·4H_2_O structures, hydrated with four water molecules, for which equilibrium fraction amounts to *x*_M_ ≥ 0.01, optimized at CAM-B3LYP/6-31++G(d,p) level,^33,34^ along with their names. The most stable geometry is shown in the box.

Although the system with four water molecules is the most complex, its computational characteristics are in the best agreement with the experimental ones. In fact, the VDE value for the thermodynamically most stable four-m23 system was calculated to be 2.34 eV (average VDE_avg_ is equal to 2.28 eV), while the experimental VDE for quadruply hydrated guanosine dianion was estimated at 2.30 eV (see Table 4).

**Table 4:** Relative Gibbs Free Energy Values (Δ*G*, in kcal/mol) and Vertical and Adiabatic Detachment Energy (VDE and ADE, in eV), Calculated for the [dGMP – 2H]^2–^·4H_2_O Systems at the CAM-B3LYP/6-31++G(d,p) Level, Characterized by Equilibrium Fraction *x*_M_ ≥ 0.01^a^

name	Δ*G*	VDE	ADE	x_M_
four-m1	1.6	2.23	1.76	0.03
four-m3	2.0	2.13	1.75	0.01
four-m4	1.1	2.14	1.76	0.06
four-m5	1.7	2.14	1.76	0.02
four-m8	1.6	2.18	1.79	0.03
four-m13	0.7	2.39	1.97	0.12
four-m14	1.1	2.17	1.79	0.06
four-m15	1.1	2.25	1.84	0.06
four-m16	0.8	2.12	1.75	0.10
**four-m23**	**0.0**	**2.34**	**1.90**	**0.38**
four-m27	1.7	2.24	1.82	0.02
four-m31	0.8	2.37	1.97	0.10
**weighted average**		**2.28**	**1.73**	
experimental values		2.30	2.05	

a Data for the dominant four-m23 geometry, as well as weighted average VDE and ADE, are bolded. Characteristics for all the obtained geometries for quadruply hydrated [dGMP – 2H]^2–^ are gathered in Table S4 in the Supplementary Information.

In summary, we proposed a general-purpose computational method to study microsolvated systems. Our approach is based on a series of MD calculations followed by clustering of similar structures. This leads to a set of geometries, which are then subjected to quantum chemical calculations enabling the experimental characteristics (such as ADE and VDE) to be predicted. We used this approach, MD/clustering/QM, to study microsolvation for the dianion of doubly deprotonated 5′-monophosphate of guanosine, a system that was recently found to be stable in the gas phase. The water clusters, [dGMP – 2H]^2–^·*n*H_2_O (*n* = 1–4), were measured with NIPE spectroscopy, and we demonstrated that the computational characteristics obtained by our MD/clustering/QM methodology are in pretty good accordance with the experimental values. As a consequence, for the first time our studies provided a “molecular structure” of the ensembles of gaseous microsolvated nucleotide clusters.

The strength of hydrogen bonds determines the specific water coordination sites in the studied clusters. Therefore, in the lowest energy configurations of the singly and doubly hydrated anion, water molecules form very strong hydrogen bonds with two charged moieties: the deprotonated amine and the phosphate group. The third water molecule in the triply hydrated anion preferentially occupies an energetically favorable site that allows it to make two hydrogen bonds with the N10 and O11 sites (for numbering, see Scheme 1). Furthermore, for the quadruply hydrated anion, the fourth water molecule interacts with the water already bonded to the guanine moiety (Figure 5, four-m23), which suggests that polarization of the bonded molecule due to hydrogen bonding with the guanine moiety makes it favorably interact with other waters or activates it for interaction with other waters. Note a similar pattern present in other low energy conformations of the quadruply hydrated anion (e.g., see four-m13 and four-m31 in Figure 5).

Water binding energies (WBEs), in terms of the free energy of cluster formation (Δ*G*_hydr-BSSE_ – difference between the free energy of cluster and the sum of the free energies of isolated monomers corrected for basis set superposition error (BSSE); see section 1.2.5 in the Supporting Information for details on the calculation of WBE), are equal to −18.0, −22.9, −26.7, and −30.5 kcal/mol for [dGMP – 2H]^2–^·H_2_O, [dGMP – 2H]^2–^·2H_2_O, [dGMP – 2H]^2–^·3H_2_O, and [dGMP – 2H]^2–^·4H_2_O, respectively (see Table S5 in the Supporting Information). Interestingly, the WBE per water molecule, Δ*G*_hydr-BSSE_/*n* (where *n* stands for the number of water molecules in a given cluster), is perfectly linearly correlated (*R*^2^ = 1.000) with the increase of VDE with the number of water molecules (see Figure S1 in the Supporting Information). For the most stable cluster of the quadruply hydrated dianion, one of the water molecules makes hydrogen bonds with another water rather than with the nucleotide moiety, which suggests that further increase in the number of waters may facilitate proton transfer (PT) from a water interacting directly with the nucleotide. Indeed, a product of such a PT process, the hydroxyl anion, can be stabilized by additional waters.^35^ Thus the proposed approach opens a route to the systematic studies of hydration effects in transition from the gas phase to bulk water. It is difficult to overestimate the importance of this issue in the DNA context, since interactions between a solute and water may qualitatively change the chemistry triggered by electron attachment in DNA based systems. The approach presented in the current work might, thus, lead to understanding of the very different sensitivity of native DNA to electrons in vacuum and under physiological conditions.

## Supporting Information Available

Experimental methods and computational details along with the corresponding references, relative Gibbs free energy values, VDE, ADE and equilibrium ratio (*x*_M_) values calculated at CAM-B3LYP/6-31++G(d,p) level, for the obtained [dGMP – 2H]^2–^·*n*H_2_O systems (*n* = 1–4), water binding energies, plot of water binding energy per water molecule vs experimental VDE increment (PDF)

## Notes

The Molecular Dynamics input and output files, as well as the Gaussian output files containing CAM-B3LYP data of optimized dianions and their corresponding anion radicals will be deposited to the zenodo.org repository, DOI: 10.5281/zenodo.5808759, upon publication.
