# Supplementary material for: Guanosine Dianions Hydrated by One to Four Water Molecules
Source: J Phys Chem Lett. 2022 Apr 5;13(14):3230–6. doi: 10.1021/acs.jpclett.2c00512 (PMC9014458; doi:10.1021/acs.jpclett.2c00512)
Supplement: Supplementary file 1 — jz2c00512_si_001.pdf [file jz2c00512_si_001.pdf]

Supporting Information

Guanosine Dianions Hydrated by One to Four Water Molecules

Samanta Makurat[1,#], Qinqin Yuan[2,5,#], Jacek Czub[3,4,#], Lidia Chomicz-Mańka[1,#], Wenjin Cao[2], Xue-Bin Wang[2*], and Janusz Rak[1*]

[1] *Faculty of Chemistry, University of Gdańsk, Wita Stwosza 63, Gdańsk 80-308, Poland*

[2] *Physical Sciences Division, Pacific Northwest National Laboratory, Richland, Washington 99352, USA*

[3] *Department of Physical Chemistry, Gdańsk University of Technology, Narutowicza 11/12, Gdańsk 80-233, Poland*

[4] *BioTechMed Center, Gdansk University of Technology, Narutowicza 11/12, 80-233, Gdansk, Poland*

[5] *Department of Chemistry, Anhui University, Hefei, Anhui, 230601, China*

# These authors contributed equally to this work.

1. Methodology

1.1 Experimental methods

The NIPE spectra were recorded using a home-made cryogenic magnetic-bottle time-of-flight (TOF) photoelectron spectrometer, coupled with an electrospray ionization (ESI) source and a temperature-variable cryogenic ion trap.[1] The $[dGMP\text{-}2H]^{2-}$ dianion and its solvated clusters were produced by spraying 0.1 mM solutions of the corresponding sodium salt in H_2O/CH_3CN (1:3) under a humidity-controlled environment. The clusters were guided by an RF quadrupole ion guide and detected by a quadrupole mass spectrometer to optimize ESI conditions to ensure stable and intense solvated cluster beams. Then, the anions were directed into the cryogenic 3D ion trap, where they were accumulated and cooled by collisions with a cold buffer gas (20% H_2 balanced in helium). In the present work, the temperature inside the ion trap was set at 20 K. The cryogenic anions were then pulsed out into the extraction zone of a TOF mass spectrometer at a 10 Hz repetition rate. Each of the desired clusters was mass-selected and maximally decelerated before being photodetached in the interaction zone with 157 nm (7.867 eV) light from an F_2 excimer laser. The laser was operated at a 20 Hz repetition rate with the ion beam off at alternating laser shots for shot-by-shot background subtraction. The distribution of the detached electrons was analyzed using a 5.2 m-long magnetic-bottle electron flight tube, and then converted into kinetic energy spectrum after calibration with the known data of I^- and $OsCl_6^{2-}$. The electron binding energy (EBE) spectra were obtained by subtracting the kinetic energy spectra from the photon energy used. The electron energy resolution ($\Delta E/E$) was about 2%, i.e., ~20 meV for 1 eV kinetic energy electrons.

1.2 Computational methods

To interpret the obtained PES spectra of the $[dGMP\text{-}2H]^{2-}{\bullet}nH_2O$ anions, quantum chemistry calculations preceded by molecular dynamics based conformational search were conducted for all solvated systems. The initial $[dGMP\text{-}2H]^{2-}$ geometry was taken from our previous study.[2] In order to identify the most important structures for the geometries generated in the MD simulation, a clustering against each of four microsolvated groups was used. Then the contributions of particular structures were computed based on the differences in their Gibbs free energies calculated quantum-chemically. Finally, the VDE and ADE values for the most represented anionic geometries were calculated and compared to the experimental data.

1.2.1 Molecular dynamics conformational search

The geometries of complexes for QM calculations were obtained from clustering the MD simulations performed with Gromacs18.[3-8] The nucleoside dianion starting structure was taken from our previous study and parametrized with parmbsc1[9] forcefield (DG3) and phosaa10

modifications (for the additional hydrogen atom) with charges calculated explicitly with Gaussian16,[10] B3LYP[11]/6-31++G(d,p).[12-14] One to four waters were placed around the nucleoside dianion randomly and the complex put into a geometric center of cubic box with side length 40 Å. Three replicas with different starting points (water orientations) were prepared. After a short steepest descent minimization (up to 5000 steps) 1 microsecond NVT simulations were performed with a timestep of 2 fs for each replica in 200 and 300 K resulting in 6 simulations for every n=1 to 4 waters of the $[dGMP\text{-}2H]^{2-}\bullet nH_2O$ systems. Note that the targeted hydrated clusters were generated from ESI solutions at ambient conditions and the rapid solvent evaporation and cryogenic cooling afford to freeze and retain conformational spaces accessible at near room temperatures.[15] A Verlet cut-off and a twin range cut-off[16] of 18 Å was used along with PBC conditions. LINear Constraint Solver (LINCS)[17,18] was used to constraint hydrogen bonds and Berendsen thermostat.[19] The center of mass translation and rotation around the center of mass were removed. Next, we clustered the last 900 ns of each of the generated MD trajectories using the gromos algorithm implemented in Gromacs 18. For that root mean square deviation (RMSD) was computed based on all atoms and the cut-offs of 0.1, 0.15, 0.2 and 0.25 Å were applied for 1, 2, 3 and 4 waters, respectively. The representative structures of each cluster were further transferred to QM calculations.

1.2.2 Quantum chemistry methodology adjustment

In order to choose an appropriate DFT level, four XC functionals were tested on the set of ten manually prepared singly solvated $[dGMP\text{-}2H]^{2-}\bullet H_2O$ dianions (manual-1 to manual-10). We compared the experimental PES spectrum with the VDE and ADE values calculated for those ten systems using the following four methods: i) B3LYP[11] and ii) CAM-B3LYP[20] both combined with the 6-31++G(d,p)[12-14] basis set. These two methods were used in our previous work on non-solvated $[dGMP\text{-}2H]^{2-}$.[2] Additionally iii) the ωB97XD[21] functional combined with the aug-cc-pvdz basis set, suggested in Wang et al. benchmark studies on multiply charged anions[22] as performing similarly well as the CAM-B3LYP functional, was tested. Finally, we also considered iv) the HSE06[23] functional combined with the aug-cc-pvdz basis set, which was found to be the best method for reproducing the experimental values of VDE in anionic silicon clusters.[24] The best agreement between experimental and calculated VDE values for the $[dGMP\text{-}2H]^{2-}\bullet H_2O$ dianions was observed for the CAM-B3LYP/6-31++G(d,p) method, which only slightly underestimates the experimental VDE (1.63 eV vs. 1.8 eV found experimentally). VDEs obtained for the remaining functional were calculated at 1.52, 1.49 and 1.42 eV for B3LYP, ωB97XD and HSE06, respectively. Thus, CAM-B3LYP/6-31++G(d,p) (gas phase) level of theory was chosen as the most reliable one for the current study.

1.2.3 Contribution of particular geometries to the equilibrated mixture of conformers

Molecular dynamics based conformational search led to quite large number of initial geometries for each microsolvated system: 35 geometries (plus set of 10 geometries prepared manually during methodology adjustment part) for $[dGMP\text{-}2H]^{2-}\bullet H_2O$, 36 geometries for $[dGMP\text{-}2H]^{2-}\bullet 2H_2O$, 31 geometries for $[dGMP\text{-}2H]^{2-}\bullet 3H_2O$ and 43 geometries for the $[dGMP\text{-}2H]^{2-}\bullet 4H_2O$ anion. VDEs calculated for the singly hydrated $[dGMP\text{-}2H]^{2-}\bullet H_2O$ system span a wide range from 1.23 to 1.74 eV. Since PES spectrum usually represents the VDE energies of the most stable anionic conformers,[25] we decided to calculate the measured characteristics for only those geometries that are the most stable thermodynamically. In a thermodynamically equilibrated system one can define the equilibrium constant K_M as:

$$K_M=[M]/[Ref]= e^{-\Delta G/RT}$$

where ΔG is the difference in Gibbs free energy between the geometry of M (e.g. any conformation of dianionic $[dGMP\text{-}2H]^{2-}\bullet nH_2O$) and the reference geometry Ref (the most stable geometry of dianionic $[dGMP\text{-}2H]^{2-}\bullet nH_2O$). Obviously, K_{Ref} is then equal to 1. The equilibrium fraction, x_M, of conformation M in the system containing n conformations can be then estimated as:[26]

$$x_M = K_M/(K_{Ref} + K_1 + K_2 + ... + K_n)$$

Considering only the structures of $x_M \geq 0.01$ for further discussion the number of conformations is reduced to 13, 7, 9 and 11 for the system solvated with 1, 2, 3 and 4 water molecules, respectively. Those the most represented conformations were discussed in the main discussion, while for full data see Table S1-S4.

1.2.4 VDE and ADE definitions

Vertical detachment energy (VDE) of $[dGMP\text{-}2H]^{2-}\bullet nH_2O$ is defined as the difference in electronic energy of dianionic system and its corresponding anion radical, both in the optimized dianion geometry, while adiabatic detachment energy (ADE) is the difference in Gibbs free energy between dianionic and anion radical systems, both in their optimal geometries. To reduce the set of VDE/ADE values to a single number, we also calculated a weighted average VDE (VDE_{avg}) and ADE (ADE_{avg}), taking into account the contribution of each conformer to the equilibrated mixture of conformers (for the complete rather than reduced data sets):

$$VDE_{avg} = \sum_{1}^{M} VDE_M \cdot x_M$$

$$ADE_{avg} = \sum_{1}^{M} ADE_M \cdot x_M$$

1.2.5 Hydration energies

For particular hydrates, hydration energies were calculated as the difference between the sum of the free energies (ΔG_{hyd}) of non-interacting monomers and that of the cluster, all calculated at the optimal geometries:

$$\Delta G_{hyd} = G([dGMP\text{-}2H]^{2-}\bullet nH_2O) - n\bullet G(H_2O) - G([dGMP\text{-}2H]^{2-})$$

where G(X) denote the free energy of species X obtained by correcting its electronic energy for zero-point vibration, thermal contributions to energy from vibrations, rotations, and translations, entropy and the pV term. These terms were determined in the harmonic oscillator-rigid rotor approximation for T = 298 K and p = 1 atm. The values of ΔG_{hyd} corrected for BSSE energy using the counterpoise procedure of Boys and Bernardi[27] are denoted $\Delta G_{hyd\text{-}BSSE}$:

$$\Delta G_{hyd\text{-}BSSE} = \Delta G_{hyd} + \text{BSSE energy}$$

2. Relative stability, VDE, ADE and water binding energy for all the obtained $[dGMP\text{-}2H]^{2-}\bullet nH_2O$ systems

Table S1. Relative Gibbs free energy values (ΔG, in kcal/mol), vertical and adiabatic detachment energy (VDE and ADE, in eV) and equilibrium fraction values (x_M) calculated for the $[dGMP\text{-}2H]^{2-}\bullet H_2O$ systems. Data for the structures of $x_M \geq 0.01$ as well as weighted average VDE and ADE bolded.

Name	ΔG	VDE	ADE	x_M
**manually prepared structures**				
manual-1	7.2	1.62	1.19	3.48E-06
manual-2	12.3	1.47	1.02	7.35E-10
manual-3	12.4	1.40	0.97	5.98E-10
manual-4	*the same as manual-1, excluded*			
manual-5	11.2	1.27	0.91	4.17E-09
manual-6	14.5	1.28	0.86	1.60E-11
manual-7	12.4	1.40	0.97	5.85E-10
manual-8	10.3	1.23	0.87	1.91E-08
**manual-9**	**2.5**	**1.63**	**1.20**	**1.08E-02**
manual-10	5.1	1.62	1.31	1.26E-04
**based on the 200K MD clustering**				
one-200-m1	7.7	1.70	1.38	1.68E-06
one-200-m2	9.4	1.64	1.34	8.74E-08
one-200-m3	10.2	1.35	1.10	2.24E-08
one-200-m4	11.4	1.63	1.33	3.23E-09
one-200-m5	12.6	1.57	1.30	3.95E-10
one-200-m6	13.8	1.33	1.10	5.39E-11
**based on the 300K MD clustering**				
**one-m1**	**2.2**	**1.59**	**1.31**	**1.69E-02**
**one-m2**	**1.6**	**1.64**	**1.33**	**4.87E-02**
one-m3	4.0	1.27	1.05	7.71E-04
one-m4	3.5	1.36	1.11	1.82E-03
**one-m5**	**2.6**	**1.65**	**1.36**	**8.53E-03**
one-m6	5.2	1.58	1.30	1.19E-04
**one-m7**	**2.0**	**1.65**	**1.33**	**2.45E-02**
one-m8	4.8	1.50	1.22	2.08E-04
**one-m9**	**2.0**	**1.36**	**1.18**	**2.55E-02**
one-m10	4.2	1.29	1.05	5.66E-04
one-m11	3.7	1.26	1.03	1.30E-03
**one-m12**	**2.6**	**1.59**	**1.31**	**8.77E-03**
one-m13	4.6	1.63	1.32	2.85E-04
**one-m14**	**1.9**	**1.36**	**0.97**	**3.11E-02**
one-m15	3.2	1.61	1.24	3.23E-03
one-m16	3.8	1.30	1.07	1.12E-03
**one-m17**	**1.6**	**1.67**	**1.35**	**4.93E-02**
**one-m18**	**2.6**	**1.65**	**1.36**	**8.55E-03**
one-m19	4.8	1.34	1.10	2.16E-04

**one-m20**	**2.0**	**1.70**	**1.38**	**2.60E-02**
one-m21	3.8	1.29	1.06	1.13E-03
one-m22	6.9	1.28	1.03	6.24E-06
**one-m23**	**0.0**	**1.70**	**1.37**	**7.14E-01**
one-m24	4.8	1.50	1.20	2.25E-04
one-m25	5.5	1.59	1.29	6.94E-05
one-m26	8.1	1.37	1.13	8.76E-07
one-m27	6.4	1.30	1.07	1.36E-05
one-m28	5.5	1.67	1.35	7.02E-05
**one-m29**	**2.3**	**1.33**	**0.97**	**1.58E-02**
**weighted average**		**1.66**	**1.34**	
experimental		1.80	1.55	

Table S2. Relative Gibbs free energy values (ΔG, in kcal/mol), vertical and adiabatic detachment energy (VDE and ADE, in eV) and equilibrium fraction values (x_M) calculated for the $[dGMP\text{-}2H]^{2-}{\bullet}2H_2O$ systems. Data for the structures of $x_M \geq 0.01$ as well as weighted average VDE and ADE bolded.

Name	ΔG	VDE	ADE	x_M
**based on the 200K MD clustering**				
two-200-m1	7.4	2.09	1.70	1.71E-06
two-200-m2	7.6	1.81	1.47	1.32E-06
two-200-m3	*the same as two-200-m2, excluded*			
two-200-m4	9.9	2.09	1.58	2.54E-08
two-200-m5	9.3	1.69	1.40	6.63E-08
two-200-m6	7.3	2.03	1.65	1.92E-06
two-200-m7	11.3	1.79	1.46	2.60E-09
**based on the 300K MD clustering**				
two-m1	2.7	1.69	1.40	5.18E-03
two-m2	2.6	1.68	1.40	5.82E-03
**two-m3**	**1.4**	**2.00**	**1.62**	**4.37E-02**
two-m4	*the same as two-m3, excluded*			
**two-m5**	**1.9**	**1.81**	**1.46**	**1.95E-02**
**two-m6**	**2.0**	**1.74**	**1.42**	**1.48E-02**
two-m7	5.9	1.37	1.15	2.16E-05
two-m8	5.4	1.68	1.40	5.49E-05
two-m9	2.8	1.75	1.45	4.36E-03
two-m10	*the same as two-m6, excluded*			
two-m11	6.0	1.80	1.50	1.72E-05
two-m12	5.4	1.66	1.39	5.20E-05
two-m13	4.6	1.36	1.13	1.90E-04
two-m14	3.1	1.73	1.46	2.39E-03
two-m15	4.3	2.08	1.63	3.22E-04
two-m16	4.6	1.98	1.60	1.93E-04
two-m17	2.5	1.65	1.37	6.26E-03
two-m18	4.3	1.99	1.61	3.30E-04
**two-m19**	**0.0**	**1.81**	**1.32**	**4.62E-01**
two-m20	5.6	1.38	1.15	3.75E-05
two-m21	5.0	1.78	1.44	9.39E-05
**two-m22**	**2.1**	**1.75**	**1.42**	**1.25E-02**
**two-m23**	**0.1**	**1.81**	**1.53**	**3.93E-01**
**two-m24**	**1.7**	**2.07**	**1.68**	**2.77E-02**
two-m25	5.5	1.73	1.43	4.65E-05
two-m26	7.4	1.38	1.12	1.78E-06
two-m27	3.8	1.70	1.29	7.13E-04
two-m28	8.1	1.85	1.50	5.16E-07
two-m29	7.5	2.02	1.43	1.52E-06
**weighted average**		**1.82**	**1.43**	
experimental		2.05	1.80	

Table S3. Relative Gibbs free energy values (ΔG, in kcal/mol), vertical and adiabatic detachment energy (VDE and ADE, in eV) and equilibrium fraction values (x_M) calculated for the $[dGMP\text{-}2H]^{2-}$•$3H_2O$ systems. Data for the structures of $x_M \geq 0.01$ as well as weighted average VDE and ADE bolded.

Name	ΔG	VDE	ADE	x_M
**based on the 200K MD clustering**				
three-200-m1	5.8	2.14	1.75	9.74E-06
three-200-m2	5.8	2.19	1.79	1.04E-05
three-200-m3	*the same as three-200-m2, excluded*			
three-200-m4	*the same as three-200-m2, excluded*			
three-200-m5	9.2	2.29	1.85	2.99E-08
three-200-m6	9.4	2.17	1.77	2.32E-08
**based on the 300K MD clustering**				
**three-m1**	**0.2**	**2.17**	**1.76**	**1.33E-01**
**three-m2**	**0.2**	**2.17**	**1.76**	**1.35E-01**
**three-m3**	**0.5**	**2.17**	**1.77**	**7.86E-02**
**three-m4**	**0.2**	**2.09**	**1.72**	**1.18E-01**
**three-m5**	**0.2**	**2.11**	**1.72**	**1.21E-01**
**three-m6**	**0.4**	**2.08**	**1.71**	**8.89E-02**
three-m7	3.3	1.73	1.44	6.56E-04
three-m8	3.1	1.83	1.45	9.26E-04
three-m9	2.3	1.79	1.48	3.53E-03
three-m10	3.8	2.24	1.80	3.12E-04
three-m11	*the same as three-m4, excluded*			
three-m12	3.9	2.26	1.82	2.68E-04
**three-m13**	**0.3**	**2.08**	**1.71**	**1.09E-01**
three-m14	4.1	1.81	1.45	1.72E-04
**three-m15**	**0.0**	**2.07**	**1.69**	**1.80E-01**
three-m16	2.4	1.82	1.50	3.06E-03
three-m17	3.1	2.09	1.70	9.57E-04
three-m18	1.8	1.90	1.55	8.50E-03
three-m19	3.2	2.10	1.71	8.64E-04
three-m20	6.5	1.96	1.60	2.97E-06
**three-m21**	**1.4**	**2.37**	**1.93**	**1.73E-02**
three-m22	7.0	1.76	1.46	1.30E-06
three-m23	4.0	2.30	1.85	2.20E-04
three-m24	5.0	1.83	1.48	3.71E-05
three-m25	5.4	1.77	1.48	2.03E-05
**weighted average**		**2.11**	**1.73**	
experimental		2.20	1.95	

Table S4. Relative Gibbs free energy values (ΔG, in kcal/mol), vertical and adiabatic detachment energy (VDE and ADE, in eV) and equilibrium fraction values (x_M) calculated for the $[dGMP\text{-}2H]^{2-}\bullet3H_2O$ systems. Data for the structures of $x_M \geq 0.01$ as well as weighted average VDE and ADE bolded.

Name	ΔG	VDE	ADE	x_M
**based on the 200K MD clustering**				
four-200-m1	10.5	2.30	1.88	7.49E-09
four-200-m2	*the same as four-200-m1, excluded*			
four-200-m3	10.4	2.32	1.88	8.74E-09
four-200-m4	10.2	2.24	1.83	1.22E-08
four-200-m5	*the same as four-200-m1, excluded*			
four-200-m6	7.8	2.56	1.95	6.69E-07
four-200-m7	7.6	2.27	1.86	1.04E-06
four-200-m8	6.8	2.29	1.87	3.67E-06
four-200-m9	7.9	2.50	1.91	6.42E-07
four-200-m10	10.5	2.16	1.77	7.43E-09
**based on the 300K MD clustering**				
**four-m1**	**1.6**	**2.23**	**1.76**	**2.52E-02**
four-m2	5.5	2.10	1.66	3.59E-05
**four-m3**	**2.0**	**2.13**	**1.75**	**1.25E-02**
**four-m4**	**1.1**	**2.14**	**1.76**	**6.29E-02**
**four-m5**	**1.7**	**2.14**	**1.76**	**2.03E-02**
four-m6	5.0	2.18	1.80	8.45E-05
four-m7	3.3	2.46	1.78	1.53E-03
**four-m8**	**1.6**	**2.18**	**1.79**	**2.58E-02**
four-m9	5.0	2.34	1.90	8.82E-05
four-m10	3.4	2.34	1.80	1.20E-03
four-m11	3.4	2.32	1.79	1.29E-03
four-m12	5.0	2.41	1.93	7.85E-05
**four-m13**	**0.7**	**2.39**	**1.97**	**1.20E-01**
**four-m14**	**1.1**	**2.17**	**1.79**	**5.81E-02**
**four-m15**	**1.1**	**2.25**	**1.84**	**6.43E-02**
**four-m16**	**0.8**	**2.12**	**1.75**	**9.98E-02**
four-m17	*the same as four-m15, excluded*			
four-m18	2.9	1.86	1.56	2.77E-03
four-m19	5.0	1.88	1.57	8.67E-05
four-m20	5.4	1.91	1.49	3.97E-05
four-m21	6.3	2.35	1.88	8.55E-06
four-m22	2.8	2.14	1.76	3.59E-03
**four-m23**	**0.0**	**2.34**	**1.90**	**3.80E-01**
four-m24	4.8	2.21	1.80	1.22E-04
four-m25	6.1	2.38	1.91	1.18E-05
four-m26	5.7	1.83	1.48	2.34E-05
**four-m27**	**1.7**	**2.24**	**1.82**	**2.22E-02**
four-m28	3.3	2.12	1.75	1.36E-03
four-m29	6.6	2.40	1.80	5.52E-06

four-m30	5.0	1.84	1.61	7.85E-05
**four-m31**	**0.8**	**2.37**	**1.97**	**9.73E-02**
four-m32	11.7	2.17	1.45	9.50E-10
four-m33	10.8	2.12	1.51	4.64E-09
**weighted average**		**2.28**	**1.87**	
experimental		2.30	2.05	

Table S5. Water binding energies (WBE) – free energy of cluster formation (ΔG_{hyd}, in kcal/mol), BSSE corrected hydration energies ($\Delta G_{hyd\text{-}BSSE}$, in kcal/mol), WBE per water molecule ($\Delta G_{hyd\text{-}BSSE}/n$, in kcal/mol) and experimental VDE increment ($\Delta VDE_{exp} = nVDE_{exp} - (n\text{-}1)VDE_{exp}$, in eV), calculated for the most stable $[dGMP\text{-}2H]^{2-} \bullet nH_2O$ systems.

N H_2O	Name	ΔG_{hyd}	$\Delta G_{hyd\text{-}BSSE}$	$\Delta G_{hyd\text{-}BSSE}/n$	ΔVDE_{exp}
1	one-m23	-18.74	-18.01	-18.01	0.50
2	two-m19	-24.74	-22.93	-11.46	0.25
3	three-m15	-29.14	-26.68	-8.89	0.15
4	four-m23	-34.27	-30.48	-7.62	0.10

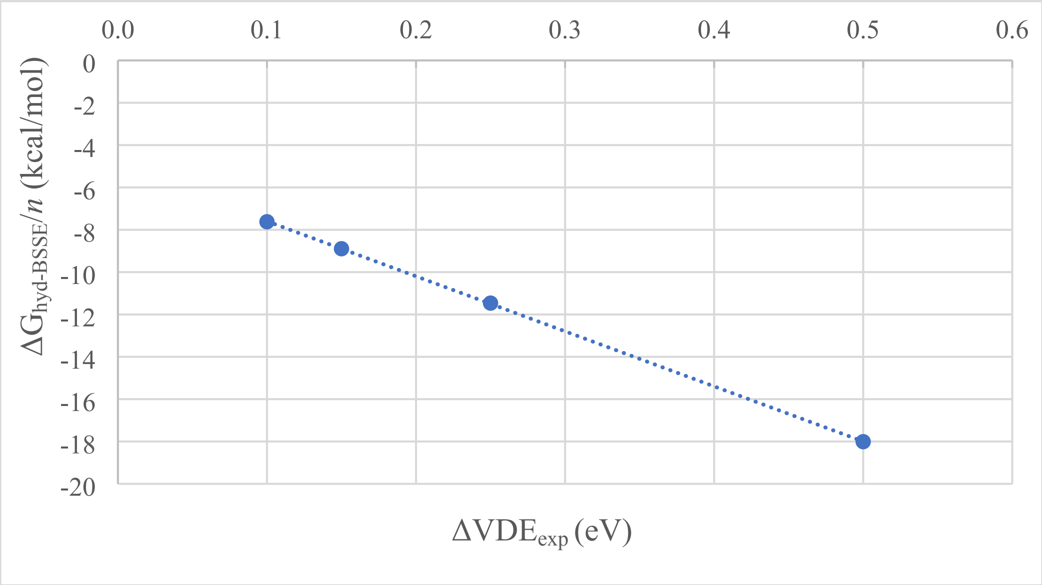

Figure S1. Water binding energy per water molecule ($\Delta G_{hyd\text{-}BSSE}/n$) dependence on experimental VDE increment (ΔVDE_{exp}).

3. References

(1) Yuan, Q.; Cao, W.; Wang, X.-B. Cryogenic and Temperature Dependent Photoelectron Spectroscopy of Metal Complexes. *Int. Rev. Phys. Chem.* **2020**, *39*, 83−108.

(2) Yuan, Q.; Chomicz-Mańka, L.; Makurat, S.; Cao, W.; Rak, J.; Wang, X.-B. Photoelectron Spectroscopy and Theoretical Investigations of Gaseous Doubly Deprotonated 2'-Deoxynucleoside 5'-Monophosphate Dianions. *J. Phys. Chem. Lett.* **2021**, *12*, 9463-9469.
(3) Abraham M. J.; Murtola T.; Schulz R.; Páll S.; Smith J. C.; Hess B.; Lindahl E. GROMACS: High Performance Molecular Simulations Through Multi-Level Parallelism from Laptops to Supercomputers. *SoftwareX* **2015**, *1*, 19-25.
(4) Pall, S.; Abraham, M. J.; Kutzner, C.; Hess, B.; Lindahl, E. Tackling Exascale Software Challenges in Molecular Dynamics Simulations with GROMACS. *Solving Software Challenges for Exascale. International Conference on Exascale Applications and Software*, EASC 2014, Stockholm, Sweden. Revised Selected Papers; Markidis, S.; Laure, E., Eds.; pp. 3−27. Springer Internat. Publ., Cham 2015.
(5) Pronk, S.; Pall, S.; Schulz, R.; Larsson, P.; Bjelkmar, P.; Apostolov, R.; Shirts, M. R.; Smith, J. C.; Kasson, P. M.; van der Spoel, D.; Hess, B.; Lindahl, E. GROMACS 4.5: A High-Throughput and Highly Parallel Open Source Molecular Simulation Toolkit. *Bioinformatics* **2013**, *29*, 845- 854.
(6) van der Spoel D.; Lindahl E.; Hess B.; Groenhof G.;. Mark A. E; Berendsen H. J. C. GROMACS: Fast, Flexible and Free. *J. Comput. Chem.* **2005**, *26*, 1701-1719.
(7) Lindahl E.; Hess B.; van der Spoel D. GROMACS 3.0: A Package for Molecular Simulation and Trajectory Analysis. *J. Mol. Model.* **2001**, *7*, 306-317.
(8) Berendsen, H. J. C.; van der Spoel D.; van Drunen R. GROMACS: A Message-Passing Parallel Molecular Dynamics Implementation. *Comput. Phys. Commun.* **1995**, *91*, 43-56.
(9) Ivani, I.; Dans, P. D.; Noy, A.; Pérez, A.; Faustino, I.; Hospital, A.; Walther J.; Andrio P.; Goñi R.; Balaceanu A.; Portella G.; Battistini F.; Gelpí J. L.; González C.; Vendruscolo M.; Laughton C. A.; Harris S. A.; Case D. A.; Orozco M. Parmbsc1: A Refined Force Field for DNA Simulations. *Nat. Methods* **2016**, *13*, 55-58.
(10) Frisch, M. J.; Trucks, G. W.; Schlegel, H. B.; Scuseria, G. E.; Robb, M. A.; Cheeseman, J. R.; Scalmani, G.; Barone, V.; Petersson, G. A.; Nakatsuji, H.; Li, X.; Caricato, M.; Marenich, A. V.; Bloino, J.; Janesko, B. G.; Gomperts, R.; Mennucci, B.; Hratchian, H. P.; Ortiz, J. V.; Izmaylov, A. F.; Sonnenberg, J. L.; Williams-Young, D.; Ding, F.; Lipparini, F.; Egidi, F.; Goings, J.; Peng, B.; Petrone, A.; Henderson, T.; Ranasinghe, D.; Zakrzewski, V. G.; Gao, J.; Rega, N.; Zheng, G.; Liang, W.; Hada, M.; Ehara, M.; Toyota, K.; Fukuda, R.; Hasegawa, J.; Ishida, M.; Nakajima, T.; Honda, Y.; Kitao, O.; Naka, H.; Vreven, T.; Throssell, K.; J.A. Montgomery, J.; Peralta, J. E.; Ogliaro, F.; Bearpark, M. J.; Heyd, J. J.; Brothers, E. N.; Kudin,

K. N.; Staroverov, V. N.; Keith, T. A.; Kobayashi, R.; Normand, J.; Raghavachari, K.; Rendell, A. P.; Burant, J. C.; Iyengar, S. S.; Tomasi, J.; Cossi, M.; Millam, J. M.; Klene, M.; Adamo, C.; Cammi, R.; Ochterski, J. W.; Martin, R. L.; Morokuma, K.; Farkas, O.; Foresman, J. B.; Fox, D. J. *Gaussian 16*, Revision C.01, Gaussian Inc., Wallingford, CT, **2016**.

(11) Becke, A. D. Density-Functional Thermochemistry. III. The Role of Exact Exchange, *J. Chem. Phys.* **1993**, *98*, 5648-52.

(12) Ditchfield, R.; Hehre, W. J.; People, J. A. Self-Consistent Molecular Orbital Methods. 9. Extended Gaussian-type Basis for Molecular-Orbital Studies of Organic Molecules. *J. Chem. Phys.* **1971**, *54*, 724.

(13) Clark, T.; Chandrasekhar J.; Spitznagel, G. W. ; Schleyer, P. v. R. Efficient Diffuse Function-Augmented Basis-Sets for Anion Calculations. 3. The 3-21+G Basis Set for 1st-Row Elements, Li-F. *J. Comput. Chem.* **1983**, *4*, 294-301.

(14) Frisch, M. J.; Pople, J. A.; Binkley, J. S. Self-Consistent Molecular Orbital Methods. 25. Supplementary Functions for Gaussian Basis Sets. *J. Chem. Phys.* **1984**, *80*, 3265-69.

(15) Zhang, H.; Cao, W.; Yuan, Q.; Zhou, X.; Valiev, M.; Kass, S. R.; Wang, X.-B. Cryogenic "Iodide-Tagging" Photoelectron Spectroscopy: A Sensitive Probe for Specific Binding Sites of Amino Acids. *J. Phys. Chem. Lett.* **2020,** *11*, 4346-4352.

(16) Páll, S.; Hess, B, A Flexible Algorithm for Calculating Pair Interactions on SIMD Architectures. *Comput. Phys. Commun.* **2013**, *184*, 2641-2650.

(17) Steinbrecher, T.; Latzer, J.; Case, D. A. Revised AMBER Parameters for Bioorganic Phosphates. *J. Chem. Theory Comput.* **2012**, *8*, 4405-4412.

(18) Hess, B.; Bekker, H.; Berendsen, H. J.; Fraaije, J. G. LINCS: A Linear Constraint Solver for Molecular Simulations. *J. Comput. Chem.* **1997**, *18*, 1463-1472.

(19) Berendsen, H. J.; Postma, J. V.; van Gunsteren, W. F.; DiNola, A. R. H. J.: Haak, J. R. Molecular Dynamics with Coupling to an External Bath. *J. Chem. Phys.* **1984**, *81*, 3684-3690.

(20) Yanai, T.; Tew, D.; Handy, N. A New Hybrid Exchange-Correlation Functional Using the Coulomb-Attenuating Method (CAM-B3LYP), *Chem. Phys. Lett.* **2004**, *393*, 51-57.

(21) Chai, J.-D.; Head-Gordon, M. Long-Range Corrected Hybrid Density Functionals with Damped Atom-Atom Dispersion Corrections. *Phys. Chem. Chem. Phys.* **2008**, *10*, 6615-20.

(22) Wang, F.; Hu, Z.; Wang, X. B.; Sun, Z.; Sun, H. Assessment of DFT Methods for the Prediction of Detachment Energies and Electronic Structures of Complex and Multiply Charged Anions. *Comput. Theor. Chem.* **2021**, *1202,* 113295.

(23) Heyd, J.; Scuseria, G. Efficient Hybrid Density Functional Calculations in Solids: The HS-Ernzerhof Screened Coulomb Hybrid Functional. *J. Chem. Phys.* **2004**, *121,* 1187-92.
(24) Wu X.; Liang X.; Du Q.; Zhao J.; Chen M.; Lin M.; Wang J.; Yin G.; Ma L.; King R. B.; von Issendorff, B. Medium-Sized (n= 14–20) Clusters: A Combined Study of Photoelectron Spectroscopy and DFT Calculations. *J. Phys.: Condens. Matter* **2018***, 30*, 354002.
(25) Svozil, D.; Jungwirth, P.; Havlas, Z. Electron Binding to Nucleic Acid Bases. Experimental and Theoretical Studies. A Review. *Collect. Czech. Chem. Commun.* **2004**, *69,* 1395-1428.
(26) Rak, J.; Piotr Skurski, P.; Simons, J.; Gutowski, M. Low-Energy Tautomers and Conformers of Neutral and Protonated Arginine. *J. Am. Chem. Soc.* **2001**, 123, 11695–11707.
(27) Boys, S. F.; Bernardi, F. The calculation of small molecular interactions by the differences of separate total energies. Some procedures with reduced errors *Mol. Phys.* **1970**, *19*, 553-566.
